# The feasibility of an implementation fidelity tool for the monitoring of a multidisciplinary lifestyle focused approach for inpatients with mental illness

**DOI:** 10.1192/j.eurpsy.2022.1594

**Published:** 2022-09-01

**Authors:** N. Den Bleijker, M. Van Schothorst, N. De Vries, P. Van Harten, J. Deenik

**Affiliations:** 1 GGz Centraal, Scientific Research Department, Amersfoort, Netherlands; 2 Maastricht University, School For Mental Health And Neuroscience, Maastricht, Netherlands

**Keywords:** Lifestyle, Fidelity, Implementation, mental illness

## Abstract

**Introduction:**

Lifestyle behaviours (e.g. physical activity and dietary habits) play a major role in the well-known premature mortality caused by poor physical health in people with mental illness. There is increasing evidence for the efficacy of lifestyle interventions on both physical and mental health, and consensus about important factors for success (e.g. targeting multiple lifestyle behaviours). However, implementation remains challenging and there is little change in clinical care. Studies that include measures of fidelity (the extent to which an intervention is implemented as intended) are able to gain insight in variations in actual implementation, which may affect intended health outcomes. However, there is currently no suitable fidelity tool for our lifestyle intervention.

**Objectives:**

A pilot study to evaluate the feasibility of a tool that assesses and monitors the implementation fidelity of a multidisciplinary lifestyle focused approach (MULTI+).

**Methods:**

MULTI+ can be tailored to various psychiatric wards and consists of 10 essential components based on scientific evidence, existing guidelines and consensus in the field of ‘lifestyle psychiatry’. We developed a tool to assess the 10 components and thereby the implementation fidelity of MULTI+. Qualitative observational data about compliance to these components are collected in 45 psychiatric wards. Adherence is converted to a gradual score (*0-50*). A higher score indicates higher fidelity.

**Results:**

Preliminary results show that the tool is feasible for use in clinical practice. Scores give insight in how various wards have implemented MULTI+.

**Conclusions:**

These outcomes can be used to further improve and understand the implementation and effectiveness of lifestyle interventions.

**Disclosure:**

No significant relationships.

